# Long noncoding RNA XIST expedites metastasis and modulates epithelial–mesenchymal transition in colorectal cancer

**DOI:** 10.1038/cddis.2017.421

**Published:** 2017-08-24

**Authors:** Dong-liang Chen, Le-zong Chen, Yun-xin Lu, Dong-sheng Zhang, Zhao-lei Zeng, Zhi-zhong Pan, Peng Huang, Feng-hua Wang, Yu-hong Li, Huai-qiang Ju, Rui-hua Xu

**Affiliations:** 1 State Key Laboratory of Oncology in South China, Collaborative Innovation Center for Cancer Medicine, Sun Yat-sen University Cancer Center, Guangzhou, China; 2 University of Texas MD Anderson Cancer Center, Houston, TX, USA

## Abstract

Tumor progression and metastasis is the main cause of death in colorectal cancer (CRC). Long noncoding RNAs (lncRNAs) are critical regulators in various diseases including human cancer. In this study, we found that lncRNA XIST was overexpressed in CRC cell lines and tissues. High expression of lncRNA XIST was associated with adverse overall survival in CRC patients. Knockdown of lncRNA XIST remarkably inhibited CRC cell proliferation, invasion, epithelial–mesenchymal transition (EMT) and CRC stem cell formation *in vitro* as well as tumor growth and metastasis *in vivo*. Further study indicated that knockdown of lncRNA XIST markedly increased the expression of microRNA-200b-3p (miR-200b-3p) that has been found to be downregulated in CRC tissues and cell lines, and luciferase activity assay indicated that lncRNA XIST could bind directly with miR-200b-3p. Moreover, knockdown of lncRNA XIST significantly reduced the expression of ZEB1, which was the direct target of miR-200b-3p, and the tumor suppressive effects caused by knockdown of lncRNA XIST could be rescued by re-expression of ZEB1 in CRC cells. Overall, our study demonstrated how lncRNA XIST regulates CRC progression and metastasis by competing for miR-200b-3p to modulate the expression of ZEB1. lncRNA XIST may be used as a biomarker to predict prognosis in CRC patients.

Globally, colorectal cancer (CRC) is one of the most common malignancies and the third leading cause of cancer-related deaths.^1^ Tumor progression and metastasis are the main causes of deaths of CRC, especially for advanced-stage patients. Despite recent progresses in the therapeutic strategies for CRC, including targeted therapies and immunotherapy, the prognosis for advanced-stage patients is still far from satisfactory. Tumor metastasis is a complex process in which the tumor cells acquire enhanced invasion abilities, and then invade into the circulation and finally colonize in the distant organ, and the critical event in this process is the accumulation of multiple genetic and epigenetic alterations that lead to the activation or inactivation of different genes.^2^ Therefore, there is an urgent need to identify the molecular mechanism of CRC progression and metastasis.

In recent years it has been recognized that the vast majority of mammalian genome are transcribed to produce noncoding RNAs (ncRNAs), among which are a group of transcripts called long noncoding RNAs (lncRNAs) that are abundantly expressed in different diseases and are involved in the regulation of tumors.^3^ lncRNAs are defined as a class of ncRNA with length >200 nucleotides with no or limited protein-coding capacity.^4^ Increasing evidences demonstrated that lncRNAs play important roles in regulating multiple biological process including cell proliferation, cell differentiation, cell invasion and chromosome inactivation.^5, 6, 7^ According to the structure and function, lncRNAs can be classified into several groups, including transcripts, antisense, circular RNAs, long intergenic ncRNAs (lincRNAs) and pseudogenes, and they can act as guides, scaffolds, tethers and decoys of other molecules, thus regulating multiple biological processes.^8, 9^ Some lncRNAs have been found to be expressed aberrantly in CRC tissues compared with normal epithelial tissue, and these lncRNAs are involved in regulating tumor phenotype by modulating the expression of their target genes.^10^

lncRNA XIST (X-inactive specific transcript) is a product of the XIST gene and the master regulator of X inactivation in mammals. Increasing studies indicated that lncRNA XIST is frequently dysregulated in multiple tumors and affects the tumor phenotypes.^11, 12, 13^ However, the role and molecular mechanism of lncRNA XIST in CRC development and metastasis is still unknown.

In this study, we found that lncRNA XIST was significantly upregulated in CRC tissues and cell lines. Overexpression of lncRNA XIST was associated with poor overall survival. Knockdown of lncRNA XIST suppressed cell proliferation, invasion, epithelial–mesenchymal transition (EMT) and stem cell formation *in vitro* as well as tumor growth and metastasis *in vivo*. Further investigation revealed that lncRNA XIST could act as a ceRNA for microRNA-200b-3p (miR-200b-3p) to modulate the expression of ZEB1.

## Results

### lncRNA XIST expression is upregulated in CRC cell lines and tissues

**Figure 1:**
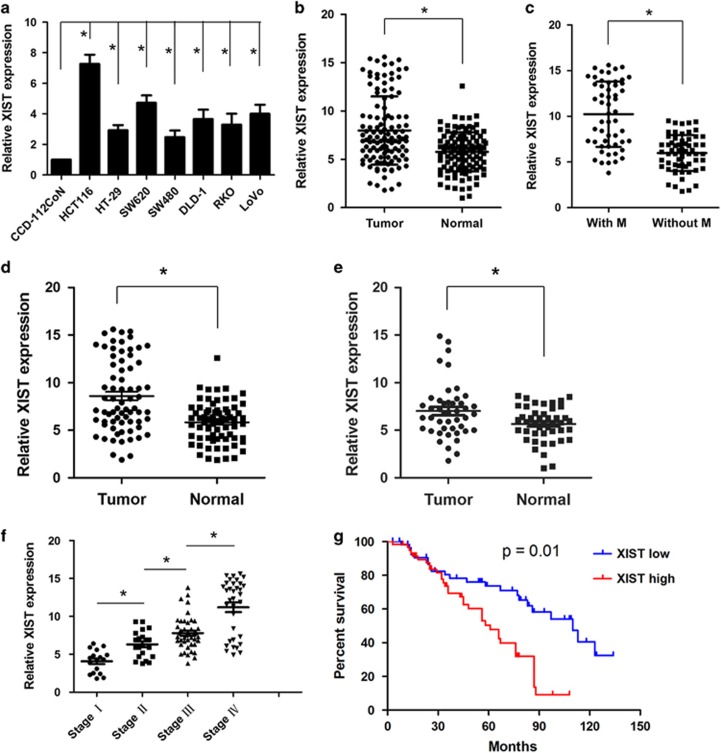
lncRNA XIST is significantly upregulated in CRC cell lines and tissues. (**a**) Relative expression level of lncRNA XIST in CRC cell lines (*P<0.05) (*n*=3 independent experiments). (**b**) Relative expression of lncRNA XIST expression in CRC tissues (*n*=115) and adjacent normal tissues (*n*=115) (*P<0.05) (*n*=3 independent experiments). (**c**) Relative expression level of lncRNA XIST in CRC tissues with (*n*=54) and without distant metastasis (*n*=61) (*P< 0.05) (*n*=3 independent experiments). (**d**) Relative expression level of lncRNA XIST in 70 male CRC tissues and the adjacent normal tissues (*P<0.05) (*n*=3 independent experiments). (**e**) Relative expression level of lncRNA XIST in 45 female CRC tissues and adjacent normal tissues (*P<0.05) (*n*=3 independent experiments). (**f**) Relative expression level of lncRNA XIST in different clinical stages (*P<0.05) (*n*=3 independent experiments). (**g**) Kaplan–Meier analysis of overall survival in CRC patients with high lncRNA XIST level (*n*=58) and low lncRNA XIST level (*n*=57) (*P*=0.01). Error bars represent the mean±S.D. values

First, the expression of lncRNA XIST was measured in CRC cell lines, compared with the normal colon epithelial cell line CCD-112CoN, and lncRNA XIST was significantly upregulated in CRC cell lines (*P<0.05, Figure 1a). lncRNA XIST expression was then measured in 115 patients with CRC tissues and paired normal tissues. Compared with adjacent normal tissues, lncRNA XIST expression was significantly upregulated in CRC tissues (*P<0.05, Figure 1b). Moreover, lncRNA XIST was higher in tumors with distant metastasis than tumors without distant metastasis (*P<0.05, Figure 1c). Previous studies have found that lncRNA plays an important role in X-chromosome inactivation, and we wondered whether gender difference exists in the expression pattern of lncRNA XIST. The result showed that in both male and female patients, the expression of lncRNA XIST was significantly higher in tumor tissues than that of adjacent normal tissues (*P<0.05, Figures 1d and e). In addition, lncRNA XIST expression was significantly increased in advanced clinical stages than that of low stages (*P<0.05, Figure 1f). To further explore the prognostic significance of lncRNA XIST in CRC, the 115 patients were classified into two groups (high expression and low expression group) according to the median level of relative lncRNA XIST expression in tumor tissues. lncRNA XIST expression was significantly associated with tumor size (*P*=0.001), histological grade (*P*=0.018), distant metastasis (*P*=0.001) and TNM stage (*P*=0.006) (Supplementary Table S1). Survival analysis was evaluated using the Kaplan–Meier method and assessed using the log-rank test. As a result, patients with higher lncRNA XIST expression had worse overall survival than those with lower lncRNA XIST expression (*P=0.01, Figure 1g). Univariate analysis indicated that other clinical and pathological parameters such as lymph node invasion (*P*=0.047), distant metastasis (*P*=0.025) and TNM stage (*P*=0.021) were also significantly associated with patients’ prognosis, but multivariate analysis demonstrated that only lncRNA XIST expression (*P*=0.039) and distant metastasis (*P*=0.033) were independent prognostic factors for CRC patients (Supplementary Table S2).

### Knockdown of lncRNA XIST inhibits cell proliferation, migration and invasion *in vitro*

**Figure 2:**
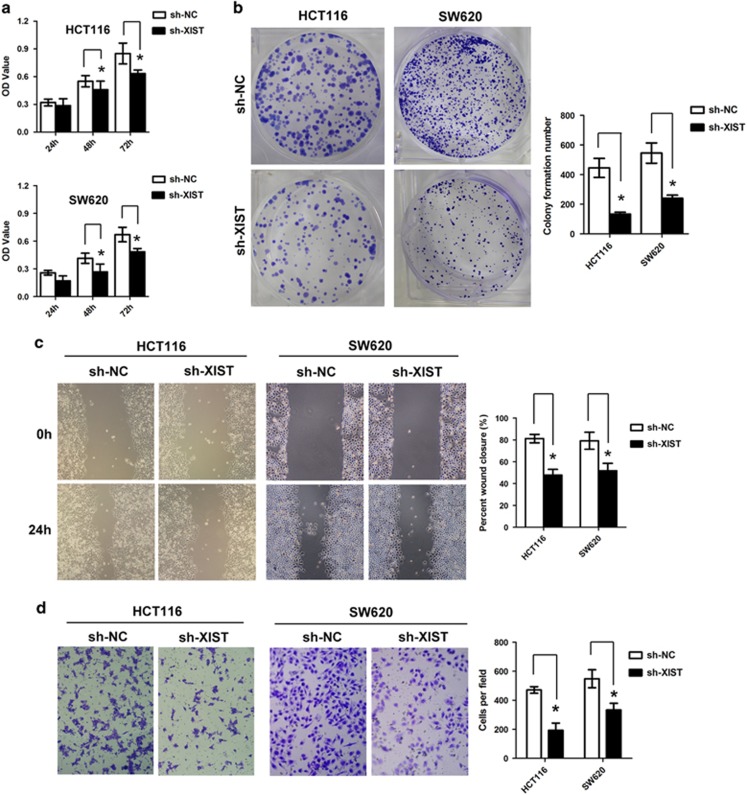
Knockdown of lncRNA XIST expression inhibits CRC cell growth, colony formation, migration and invasion *in vitro*. (**a**) Knockdown of lncRNA XIST inhibited cell proliferation as indicated by CCK-8 assays in HCT116 and SW620 cells (*P<0.05) (*n*=3 independent experiments). (**b**) Knockdown of lncRNA XIST inhibited colony formation as demonstrated by colony formation assays in HCT116 and SW620 cells (*P<0.05) (*n*=3 independent experiments). (**c**) Knockdown of lncRNA XIST inhibited cell migration in HCT116 and SW620 cells as indicated by wound healing assays (*P<0.05) (*n*=3 independent experiments). (**d**) Knockdown of lncRNA XIST inhibited cell invasion in HCT116 and SW620 cells as demonstrated by transwell assays (*P<0.05) (*n*=3 independent experiments). Error bars represent the mean±S.D. values

Having demonstrated that lncRNA XIST is involved in CRC progression, we wondered whether lncRNA XIST is required for maintenance of malignant phenotype of CRC cells. Knockdown of lncRNA XIST resulted in significant reduction of lncRNA XIST level in HCT116 and SW620 cells. CCK-8 assay showed that knockdown of lncRNA XIST markedly inhibited cell proliferation rate in HCT116 and SW20 cells (*P<0.05, Figure 2a). Colony formation assay indicated that knockdown of lncRNA XIST significantly suppressed colony formation in HCT116 and SW620 cells (*P<0.05, Figure 2b). Moreover, wound healing assay showed a significant reduction of cell migration after knockdown of lncRNA XIST in HCT116 and SW620 cells (*P<0.05, Figure 2c), and transwell assay indicated that knockdown of lncRNA XIST inhibited cell invasion of HCT116 and SW620 cells (*P<0.05, Figure 2d).

### Knockdown of lncRNA XIST inhibits EMT and stem cell formation in CRC cells

**Figure 3:**
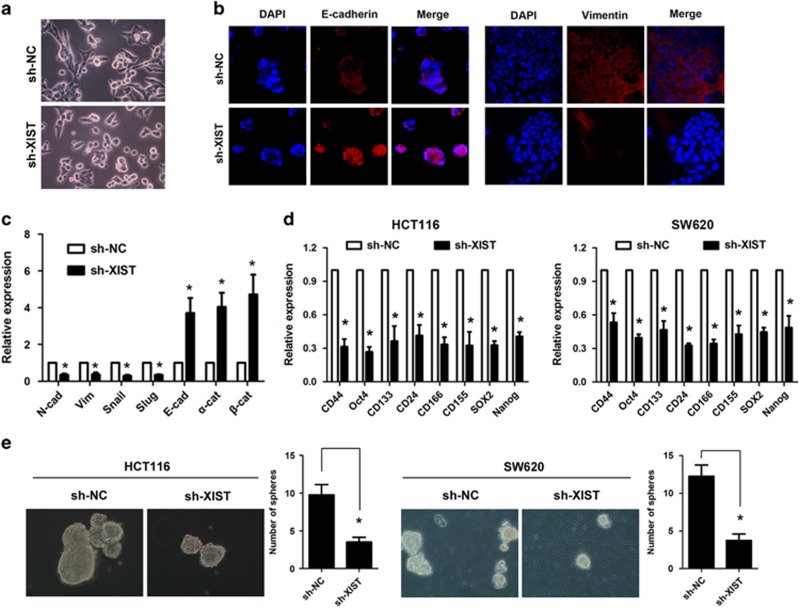
Knockdown of lncRNA XIST inhibits EMT and stem cell formation in CRC cells. (**a**) HCT116 cells underwent morphological change from a spindle shape to a rounded or cobblestone-like shape upon knockdown of lncRNA XIST. (**b**) Knockdown of lncRNA XIST significantly increased the expression of E-cadherin, whereas it decreased the expression of N-cadherin in SW620 cells as demonstrated by immunofluorescence. (**c**) Real-time PCR analysis showed that knockdown of lncRNA XIST significantly reduced the expression of mesenchymal markers (N-cadherin, Vimetin, Snail, Slug), whereas it increased the expression of epithelial markers (E-cadherin, *α*-catenin, *β*-catenin) (*P<0.05) (*n*=3 independent experiments). (**d**) Real-time PCR analysis showed knockdown of lncRNA XIST could markedly decrease the expression of many stemness-associated genes (Nanog, Oct-4 and SOX2) and surface antigens associated with cancer stem cells (CD24, CD44, CD133, CD155 and CD166) (*P<0.05) (*n*=3 independent experiments). (**e**) Knockdown of lncRNA XIST significantly reduced the sphere formation in HCT116 and SW620 cells (*P<0.05). Error bars represent the mean±S.D. values

It has been reported that EMT is a critical step of tumor metastasis. We therefore investigated whether lncRNA XIST could modulate EMT in CRC cells. The results indicated that HCT116 cells underwent morphological change from a spindle shape to a rounded or cobblestone-like shape upon knockdown of lncRNA XIST (Figure 3a), and knockdown of lncRNA XIST could significantly increase the expression of E-cadherin but decrease the expression of N-cadherin in SW620 cells as demonstrated by immunofluorescence (Figure 3b). Moreover, real-time PCR analysis also confirmed that knockdown of lncRNA XIST significantly reduced the expression of mesenchymal markers (N-cadherin, Vimetin, Snail, Slug) but increased the expression of epithelial markers (E-cadherin, *α*-catenin, *β*-catenin) (*P<0.05, Figure 3c). Furthermore, real-time PCR analysis showed knockdown of lncRNA XIST could markedly decrease the expression of many stemness-associated genes (Nanog, Oct-4 and SOX2) and surface antigens associated with cancer stem cells (CD24, CD44, CD133, CD155 and CD166) (*P<0.05, Figure 3d). In addition, knockdown of lncRNA XIST significantly reduced the sphere formation in HCT116 and SW620 cells (*P<0.05, Figure 3e).

### Knockdown of lncRNA XIST inhibits tumorigenesis and metastasis *in vivo*

**Figure 4:**
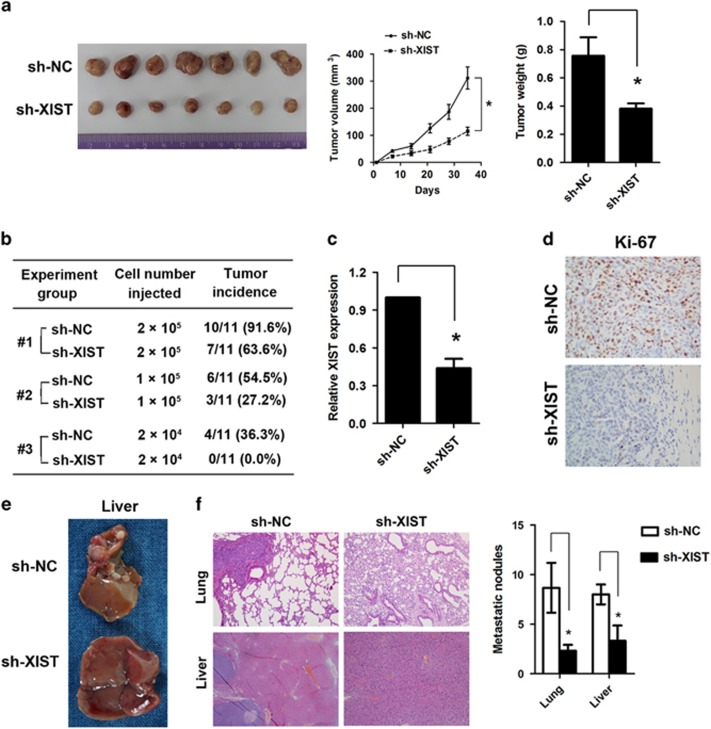
Knockdown of lncRNA XIST expression inhibits tumor growth and metastasis *in vivo*. (**a**) Knockdown of lncRNA XIST expression significantly inhibited tumor growth of HCT116 cells in nude mouse model. The tumor volume and tumor weight formed by HCT116 sh-XIST cells was significantly less than that of HCT116 sh-NC cells; the mean tumor volume was 356and 743 mm^3^ for the HCT116/sh-XIST and HCT116/sh-NC groups, respectively, and the mean tumor weight was 0.75 and 0.38 g for the HCT116/sh-XIST and HCT116/sh-NC groups, respectively (*P<0.05). (**b**) Of the mice, 91.6% developed tumors when injected with 2.0 × 10^5^HCT116 sh-NC cells. The tumor incidence was 63.6% when mice were injected with 2.0 × 10^5^ HCT116 sh-XIST cells (group 1); the tumor incidence was 54.5% when mice were injected with 1.0 × 10^5^ sh-NC cells; the tumor incidence was 27.2% when mice were injected with 1.0 × 10^5^ cells in the sh-XIST group (group 2); the tumor incidence was 36.3% and 0% when the mice were injected with 1.0 × 10^4^ cells of sh-NC and sh-XIST, respectively (group 3). (**c**) Real-time PCR analysis showed that knockdown of lncRNA XIST significantly decreased the expression of lncRNA XIST in tumor tissues taken from the nude mice (*P<0.05). (**d**) Immunohistochemistry analysis showed that knockdown of lncRNA XIST significantly reduced Ki-67 expression. (**e**) Knockdown of lncRNA XIST significantly reduced the macrometastases in the liver. Of the 11 mice in the sh-NC group, 6 formed macroscopic liver metastases, whereas only 1 of 11 mice in the sh-XIST group formed macroscopic liver metastases. (**f**) The mean micro lung metastatic nodules were 2.6 and 7.9 for the HCT116/sh-XIST and HCT116/sh-NC groups, respectively, and the mean micro liver metastatic nodules were 1.3 and 6.5 for the HCT116/sh-XIST and HCT116/sh-NC groups, respectively (*P<0.05). Error bars represent the mean±S.D. values

Having demonstrated the biological effect of lncRNA XIST *in vitro*, we wondered whether lncRNA XIST could regulate tumorigenesis and metastasis *in vivo*. To test the effect of lncRNA XIST on tumorigenesis, cells (HCT116/sh-XIST and HCT116/sh-NC) were subcutaneously injected into the flank of nude mice, and the tumor volume was measured every week. At the end of the experiment, the mice were killed and the tumors were dissected out. The results showed that the volume and weight of tumors formed by HCT116/sh-XIST cells were obviously less than that formed by HCT116/sh-NC cells. The mean tumor volume was 356 and 743 mm^3^ for the HCT116/sh-XIST and HCT116/sh-NC groups, respectively, and the mean tumor weight was 0.75 and 0.38 g for the HCT116/sh-XIST and HCT116/sh-NC groups, respectively (*P<0.05, Figure 4a). Moreover, the results demonstrated that >90% of the mice developed tumors when injected with 2.0 × 10^5^ HCT116 sh-NC cells, whereas the tumor incidence reduced to 63.6% when mice were injected with the same number of HCT116 sh-XIST cells (group 1); the tumor incidence was 54.5% in the control group when the mice were injected with 1.0 × 10^5^ cells, and the tumor incidence reduced to 27.2% in the sh-XIST group (group 2). Similarly, knockdown of lncRNA XIST dramatically reduced the tumor incidence from 36.3 to 0% when the mice were injected with 1.0 × 10^4^ cells (group 3) (Figure 4b). The real-time PCR analysis confirmed the knockdown effect of lncRNA XIST *in vivo* (*P<0.05, Figure 4c), and immunohistochemistry (IHC) analysis showed that knockdown of lncRNA XIST significantly reduced Ki-67 expression (Figure 4d). To explore the effect of lncRNA XIST on tumor metastasis, cells (sh-NC or sh-XIST, 2 × 10^6^cells/mouse) were injected into the tail vein of nude mice, and after 6 weeks the mice were killed and the lung and liver were exercised. Both the sh-NC and the sh-XIST groups did not form macroscopic metastases in the lung, and 6 of 11 mice in the sh-NC group formed macroscopic liver metastases, whereas only 1 of 11 mice in the sh-XIST group formed macroscopic liver metastases (Figure 4e). Compared with those injected with HCT116/sh-NC cells, mice injected with HCT116/sh-XIST cells formed significantly less micrometastases in the lung and liver. The mean micro lung metastatic nodules were 2.6 and 7.9 for the HCT116/sh-XIST and HCT116/sh-NC groups, respectively, and the mean micro liver metastatic nodules were 1.3 and 6.5 for the HCT116/sh-XIST and HCT116/sh-NC groups, respectively (*P<0.05, Figure 4f).

### lncRNA XIST acts as a ceRNA for miR-200b-3p to modulate ZEB1 expression in CRC cells

**Figure 5:**
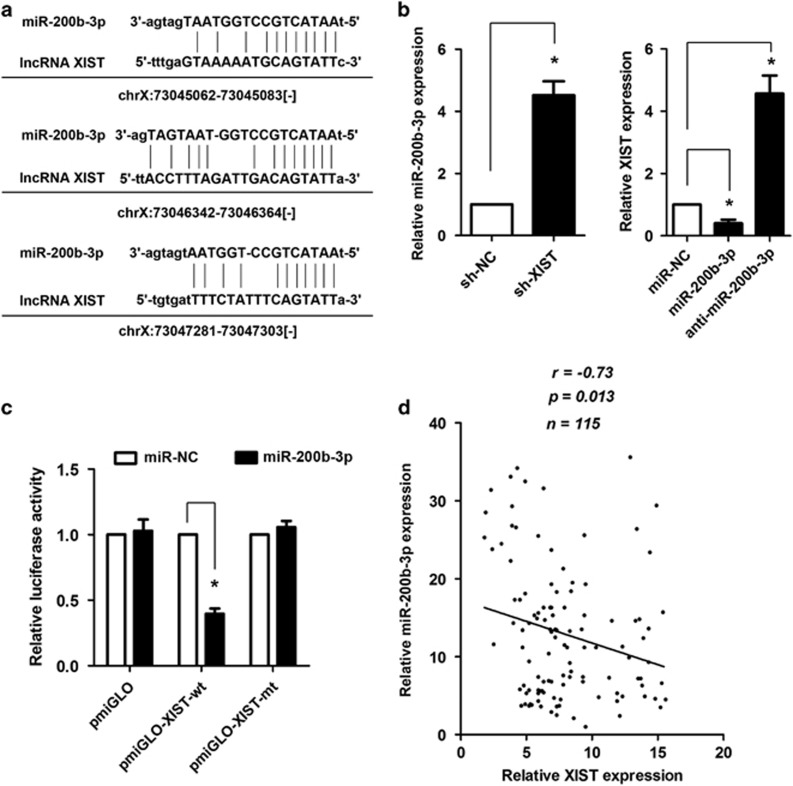
Reciprocal repression between lncRNA XIST and miR-200b-3p. (**a**) Schematic representation of the predicted target site for miR-200b-3p in lncRNA XIST. (**b**) Knockdown of lncRNA XIST increased miR-200b-3p expression in HCT116 cells; ectopic miR-200b-3p expression decreased lncRNA XIST expression, and inhibition of miR-200b-3p increased lncRNA XIST expression (*P<0.05). (**c**) Luciferase reporter assay in human embryonic kidney (HEK) 293T cells, co-transfected with the reporter plasmid (or the corresponding mutant reporter) and the indicated miRNAs. miR-200b-3p significantly decreased the luciferase activity in XIST-wt but not in XIST-mt (*P<0.05). (**d**) The expression of lncRNA XIST was inversely correlated with the expression level of miR-200b-3p in CRC tissues (*r*=−0.73, *P=0.013). Error bars represent the mean±S.D. values

**Figure 6:**
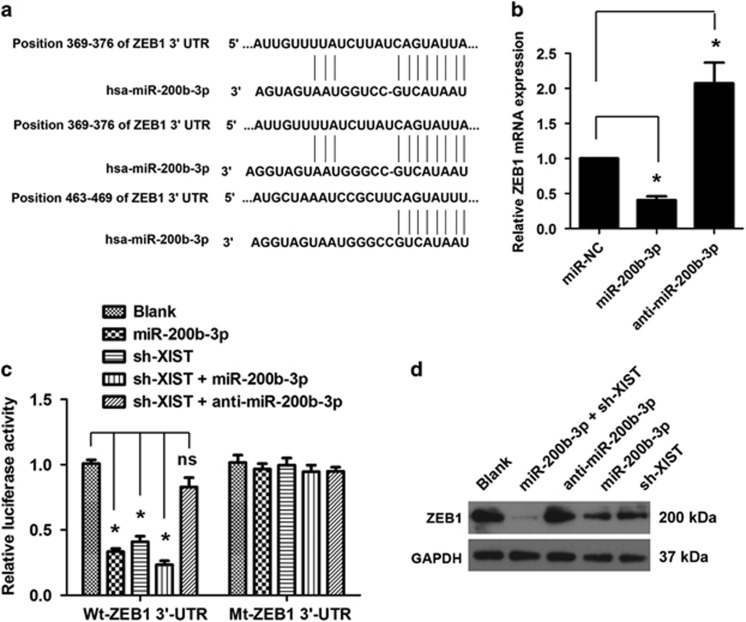
miR-200b-3p directly targets ZEB1 in CRC cells. (**a**) Schematic representation of the predicted target site for miR-200b-3p in the ZEB1 3′-UTR. (**b**) The ectopic expression of miR-200b-3p significantly decreased whereas inhibition of miR-200b-3p increased the expression of ZEB1 mRNA level in CRC cells (*P<0.05). (**c**) Luciferase activity assay in the wild type and mutant type when transfected with different vectors (*P<0.05). (**d**) ZEB1 protein level in HCT116 cells following ectopic expression of miR-200b-3p and/or knockdown of lncRNA XIST and inhibition of miR-200b-3p. Error bars represent the mean±S.D. values

Recent studies have proposed that lncRNAs may participate in the ceRNA regulatory network. By using the online software program Starbase v2.0 (http://starbase.sysu.edu.cn/), we found that lncRNA XIST formed complementary base pairing with miR-200b-3p (Figure 5a). Knockdown of lncRNA XIST could significantly increase the expression of miR-200b-3p level in CRC cell lines HCT116 (*P<0.05, Figure 5b), and ectopic expression of miR-200b-3p could obviously reduce the expression of lncRNA XIST, whereas inhibition of miR-200b-3p could increase the expression of lncRNA XIST in CRC cells (*P<0.05, Figure 5b). To confirm the direct binding relationship between lncRNA XIST and miR-200b-3p, a luciferase activity assay was performed. We found that miR-200b-3p mimics markedly reduced the luciferase activities of pmirGLO-XIST-wt. However, no obvious reduction was observed in cells transfected with miR-200b-3p mimics and pmirGLO-XIST-wt (*P<0.05, Figure 5c). In addition, an inverse correlation was found between the expression of lncRNA XIST and miR-200b-3p in CRC tissues (*r*=-0.73, *P=0.013, Figure 5d). We then explored the genes that were potentially regulated by miR-200b-3p. As lncRNA XIST could modulate EMT of CRC cells, we focused on *ZEB1*, one of the genes that was reported to be involved in EMT. As shown in Figure 6a, there are several miR-200b-3p-binding sites in the 3′-UTR of ZEB1. To our interest, ectopic expression of miR-200b-3p could decrease whereas inhibition of miR-200b-3p could increase the expression of ZEB1 mRNA level in CRC cells (*P<0.05, Figure 6b). Luciferase activity assay showed that ectopic expression of miR-200b-3p and/or knockdown of lncRNA XIST could significantly decrease the luciferase activity of the wild-type ZEB1 3′-UTR, but not of the mutant type ZEB1 3′-UTR; moreover, the reduced luciferase activity caused by lncRNA XIST knockdown could be restored by inhibition of miR-200b-3p (*P<0.05, Figure 6c). Western blot analysis indicated that ectopic expression of miR-200b-3p and/or knockdown of lncRNA XIST significantly reduced the protein level of ZEB1, whereas inhibition of miR-200b-3p increased the protein level of ZEB1 in CRC cells (Figure 6d).

### The miR-200b-3p/ZEB1 axis mediated the lncRNA XIST’ oncogenic effect in CRC cells

**Figure 7:**
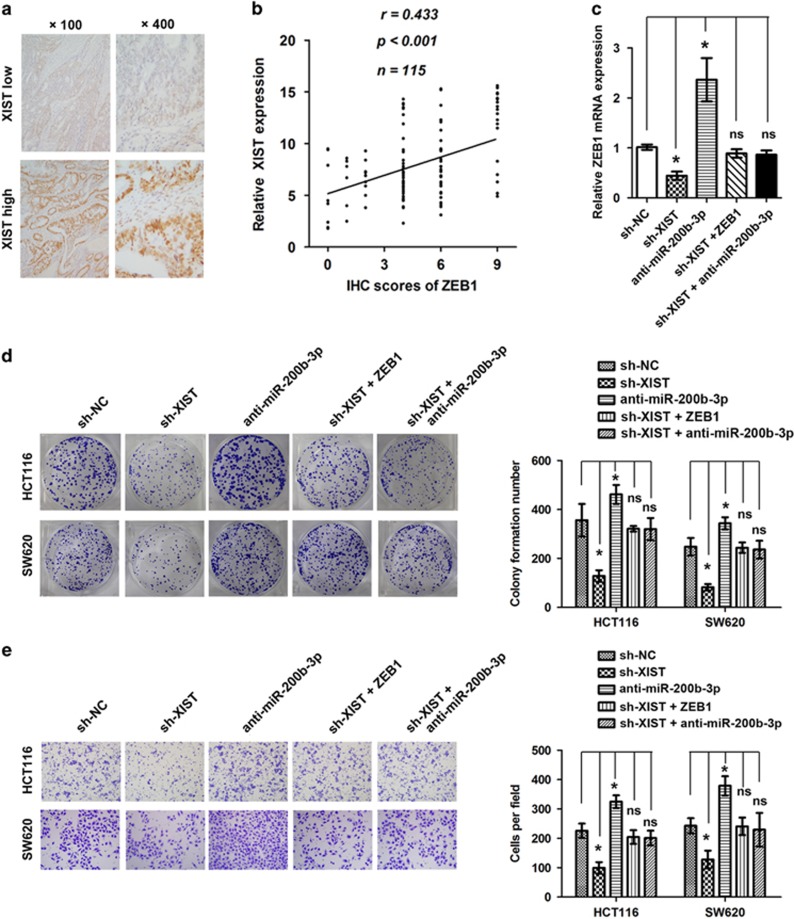
ZEB1 expression mediated the biological effects exerted by lncRNA XIST. (**a**) The expression of ZEB1 in CRC tissues detected by IHC. (**b**) The correlation between the expression of lncRNA XIST and ZEB1 (*r*=0.433, *P<0.001). (**c**) ZEB1 mRNA level following treatment of different vectors (*P<0.05). (**d**) Colony formation assay following treatment of different vectors (*P<0.05) (*n*=3 independent experiments). (**e**) Cell invasion following treatment of different vectors (*P<0.05) (*n*=3 independent experiments). Error bars represent the mean±S.D. values

IHC analysis showed that ZEB1 was overexpressed in CRC tissues (Figure 7a), and a positive correlation was observed between the expression of ZEB1 and lncRNA XIST (*r*=0.433, *P<0.001, Figure 7b). Knockdown of lncRNA XIST could significantly reduce the mRNA level of ZEB1 in CRC cells, and the reduced level of ZEB1 mRNA induced by lncRNA XIST knockdown could be restored by ectopic expression of ZEB1 or inhibition of miR-200b-3p (*P<0.05, Figure 7c). What’s more, the suppression of tumor phenotype by knockdown of lncRNA XIST could be restored by ectopic expression of ZEB1 or inhibition of miR-200b-3p (*P<0.05, Figures 7d and e).

## Discussion

Mounting evidences showed that ncRNAs play important role in tumor pathology and might be used as diagnostic and therapeutic target. For example, nuclear-enriched abundant transcript 1 can function as a diagnostic and prognostic biomarker in CRC;^14^ HOTAIR is a negative prognostic factor not only in primary tumors, but also in the blood of CRC.^15^ Zhang *et al.*^16^ found that lncRNA CASC11 can interact with hnRNP-K and activate the WNT/*β*-catenin pathway to promote growth and metastasis in CRC. Our previous study showed that knockdown of lncRNA XIST inhibited gastric cancer progression and metastasis through modulating the expression of EZH2.^17^ However, the role and molecular mechanism of lncRNA XIST in CRC remains unknown.

In this study, we found that lncRNA XIST expression was upregulated in CRC tissues than adjacent normal tissues. High lncRNA XIST expression was significantly associated with tumor size, lymph node invasion and clinical stage. Moreover, upregulation of lncRNA XIST was correlated with poor overall survival and might be used as an independent prognostic indicator for CRC patients. These results implicated that lncRNA XIST plays an important role in CRC progression. In accordance with our results, it has been found that lncRNA XIST plays an important role in different tumor types. For example, lncRNA XIST regulates PTEN expression by sponging miR-181a and promotes hepatocellular carcinoma progression;^18^ in another study, it was found that lncRNA XIST could promote pancreatic cancer proliferation through miR-133a/EGFR.^19^ However, other studies indicated that loss of lncRNA XIST is essential for some malignancies. For instance, Yildirim *et al.*^20^ reported that XIST deletion could induce hematologic cancer in female mice. Huang *et al.*^21^ demonstrated that XIST functions as a tumor suppressor in breast cancer. It seems that XIST gene is upregulated in a variety of non-sex-related tumors in both humans and mice, whereas it might be lost in some female cancers. The underlying mechanism for gender difference in XIST expression is complicated and needs further exploring. One exploration is that certain genes may be oncogenic in one cell/tissue whereas tumor suppressive in another, depending on the tumor type and cellular context.

Considering the function aspect, we found that knockdown of lncRNA XIST inhibited CRC cell proliferation and migration *in vitro*. Our data indicated that lncRNA XIST is involved in the regulation of EMT, and previous studies have demonstrated that EMT is an important step in tumor progression and metastasis.^22, 23^ More importantly, we found that lncRNA XIST could affect the stemness of CRC cells, and this is in accordance with previous studies that have demonstrated that cancer stem cells are a key source of cancer metastasis and progression, and a direct link existed between the EMT and the gain of epithelial stem cell properties.^24, 25^ These results indicated that lncRNA XIST regulated EMT and stem cell properties, leading to altered proliferation and invasion abilities, and finally affecting tumor growth and metastasis.

A growing number of studies have suggested the existence of a widespread interaction network involving ceRNAs, in which the ncRNAs can regulate miRNAs by binding to and titrating them off their binding sites on protein coding messengers. For example, lncRNA HULC is upregulated in liver cancer, and HULC overexpression can promote tumor progression in part through its inhibitory effects on the expression and activity of miR-372.^26^ Similar reports indicated that H19 functions as ceRNA to modulate let-7 and promotes breast cancer stem cell maintenance.^27^ Liu *et al.*^28^ revealed a reciprocal repression between loc285194 and miR-211 in colon cancer. In this study, we used bioinformatics databases (Starbase v2.0, miRcode and RNAhybrid) and identified several miRNAs that might interact with lncRNA XIST. To our interest, miR-200b-3p was one of the miRNAs that could be potentially regulated by lncRNA XIST, and miR-200b-3p has been found to be downregulated in CRC and other tumors.^29, 30^ The regulating relationship between miR-200b-3p and lncRNA XIST was confirmed by the following evidences: (1) miR-200b-3p expression was markedly increased upon knockdown of lncRNA XIST; (2) ectopic expression of miR-200b-3p obviously decreased the expression of lncRNA XIST, whereas inhibition of miR-200b-3p increased the expression of lncRNA XIST; (3) an inverse association existed between the expression level of lncRNA XIST and miR-200b-3p in CRC tissues; (4) luciferase activity assay confirmed that miR-200b-3p could directly bind to the 3′-end of lncRNA XIST. Similar to our results, a recent report indicated that lncRNA XIST functions as a molecular sponge for miR-139-5p in hepatocellular carcinoma.^31^ This implies that lncRNA XIST may interact with different miRNAs depending on tumor types.

ZEB1 is an important regulator of EMT and cell invasion in different tumor types.^32, 33^ In this study, we found that ZEB1 was directly targeted by miR-200b-3p. lncRNA XIST could indirectly regulate ZEB1 expression by way of sponging to miR-200b-3p. The tumor suppressive effect of lncRNA knockdown could be restored by re-expression of ZEB1 in CRC cells. In addition, we found that ZEB1 was upregulated and positively associated with lncRNA XIST expression in CRC. This is in line with previous reports that indicated ZEB1 confers EMT, stem-like properties and stimulates tumor progression.^34, 35^ It has been reported that some lncRNAs can influence transcription at enhancers and promoters. We determined whether lncRNA XIST could modulate the transactivation of ZEB1 mRNA. The promoter of ZEB1 was cloned upstream of a luciferase reporter gene, and the resultant construct was co-transfected with lncRNA XIST in CRC cells. However, the result showed that lncRNA XIST did not affect the transactivation of the ZEB1 promoter (data not shown), implying that lncRNA XIST might regulate ZEB1 after it is transcribed. Taken together, these data showed that lncRNA XIST functioned as a ceRNA for miR-200b-3p to regulate ZEB1 expression.

**Figure 8:**
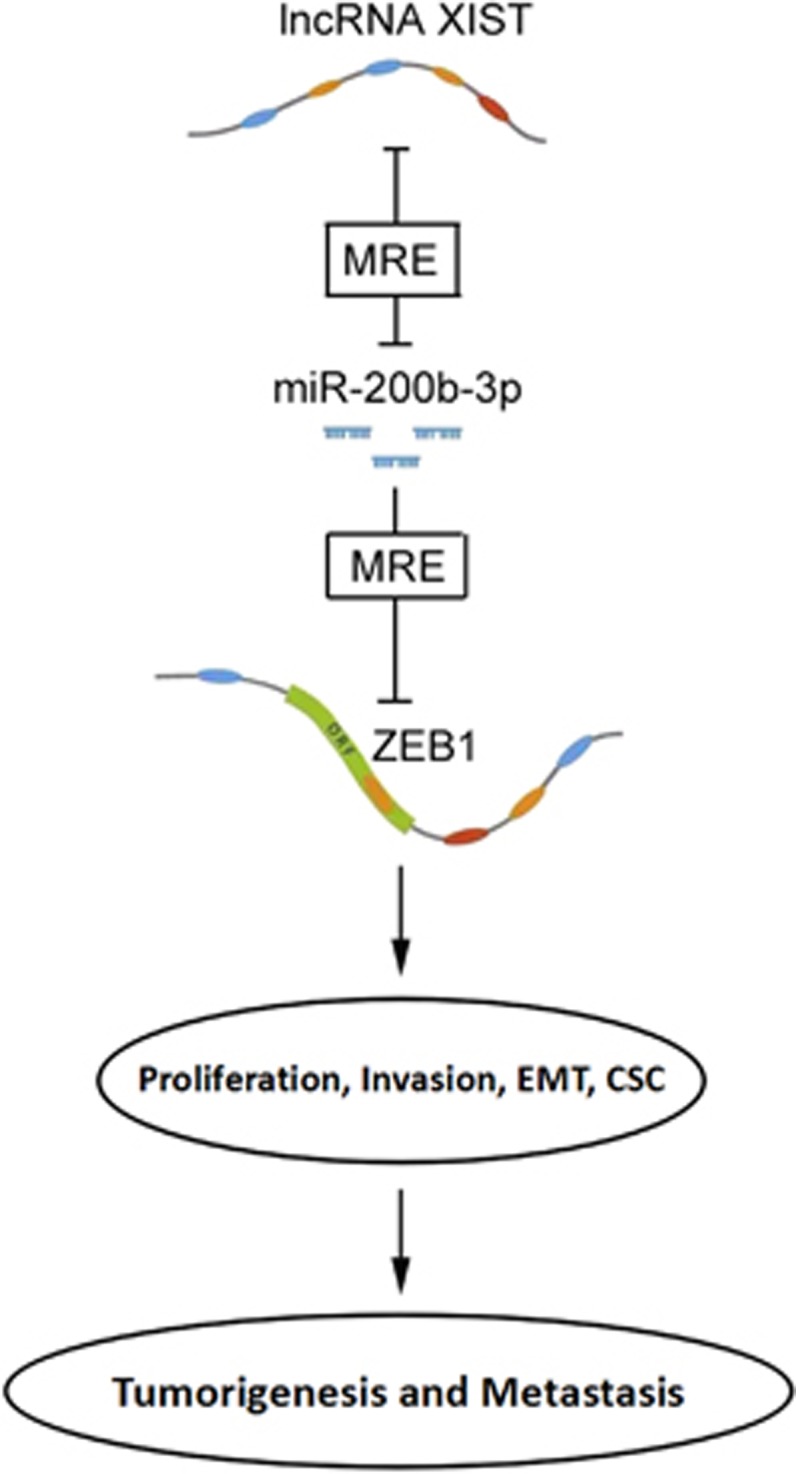
The functional model underlying the mechanism of lncRNA XIST on CRC tumorigenesis and metastasis. lncRNA XIST sponging to miR-200b-3p through the MRE (microRNA response element); it thus acts as a ceRNA to regulate the expression of ZEB1 and modulate the proliferation, invasion, EMT and stem cell properties *in vitro* and tumorigenesis and metastasis *in vivo*

In conclusion, we provided the first evidence that lncRNA XIST was overexpressed in CRC tissues, and lncRNA XIST upregulated the miR-200b-3p target gene ZEB1 by way of competitively ‘sponging’ miR-200b-3p, and then promoted cell proliferation, invasion, EMT and stem cell formation *in vitro* as well as tumorigenesis and metastasis *in vivo* (Figure 8). lncRNA XIST might be used as a prognostic biomarker for CRC patients. As lncRNA XIST loss might induce hematopoietic malignancies and other X-chromosome gene activation events that may cause gene-dosage-associated pathologies in females,^20^ the application of targeting XIST should be individualized in CRC patients, it might be more feasible in male CRC patients and should be used with caution in female patients.

## Materials and methods

### Human tissue samples

CRC specimens and adjacent normal tissues were obtained from 115 patients who received surgery in SUSYCC from May 2008 to July 2012. All the samples were confirmed by pathologists. None of the patients receive any treatment before surgery. Each patient was returned for follow-up visit with an interval of 3 months. The clinical and pathological characteristics were obtained from patients’ history record. Overall survival time was defined as the date of operation to the date of death or last contact. This study has been approved by the institutional ethics review board of Sun Yat-sen University Cancer Center (SYSUCC) (Guangzhou, China) and all patients provided written informed consent before participating in this study.

### Cell lines

Human CRC cell lines including HCT116, HT-29, SW620, SW480, DLD-1, RKO, LoVo, the normal colon epithelial cell line CCD-116CoN and the human embryonic kidney (HEK) 293T cell were obtained from the American Type Culture Collection (Manassas, VA, USA). Cells were cultured and stored according to the provider’s instructions, and were routinely authenticated every 6 months by cell morphology monitoring and growth curve analysis.

### RNA extraction and real-time PCR analysis

Total RNA was extracted from tissues and cells with Trizol reagent (Takara, Otsu, Japan) according to the manufacturer’s instructions. NanoDrop ND-2000 spectrophotometer (Thermo Scientific, Wilmington, DE, USA) was used to measure the RNA concentration and purity. The reverse transcription for lncRNA XIST and ZEB1 was performed with the High-Capacity cDNA Reverse Transcription Kit (Applied Biosystems, Foster City, CA, USA). RNA (2 *μ*g) from each tissue and cell sample was used to synthesize cDNA using a reverse transcription kit (Takara). Real-time PCR was performed using SYBR Green Real-Time PCR MasterMix (Toyobo, Osaka, Japan), and GAPDH was used as the reference. The primers used for the real-time PCR are listed in Supplementary Table S3. The All-in-One miRNA qRT-PCR Detection Kit (GeneCopoeia, Rockville, Montgomery, USA) was used to detect the miR-200b-3p level according to the provider’s instructions; U6 small RNA was used as the positive control. Real-time PCR was performed with the Bio-Rad CFX96 qPCR system (Hercules, CA, USA) and fold changes were determined using the relative quantification 2^−△△CT^ method.

### Lentivirus production and infection

The details for constructing stable knockdown cells of lncRNA XIST can be found in our previously published study.^17^

### Cell transfections

Cell transfections were performed according to the method described previously.^36^ Briefly, the cells were cultured in a 6-well plate the day before transfection. Cell transfections were performed using Lipofectamine 2000 (Invitrogen, Carlsbad, CA, USA) with a final concentration of 50 nM and collected for assays after 48 h. The miR-200b-3p mimic, miR-200b-3p inhibitor and negative control (NC) oligonucleotides were purchased from Ribobio (Guangzhou, China). To rescue the expression of ZEB1, cells were co-transfected with a pcDNA3.1-ZEB1 plasmid that contained the coding sequences but lacked the 3′-UTR of ZEB1.

### Cell proliferation assay

Cell proliferation was performed using CCK-8 assays. Briefly, 1 × 10^3^ cells were cultured in a 96-well plate at 37 °C. Plates were incubated at 37 °C for 2 h after each well was added with 10 *μ*l CCK-8 solution. Then, the spectrophotometric absorbance was measured at 570 nm for each sample. All the experiments were performed in triplicate and repeated 3 times, and the mean value was calculated.

### Cell colony formation assay

Cells were trypsinized and suspended in RPMI-1640 medium (GIBCO, Grand Island, NE, USA) with 10% FBS, the cells were then seeded in 6-well plates in triplicate and cultured in a humidified atmosphere containing 5% CO_2_ at 37 °C for 14 days. Cell colonies were washed with PBS, fixed with methanol for 30 min and stained with 0.1% crystal violet (1 mg/ml) for 20 min. Colonies containing >50 cells were counted, and the mean colony numbers were calculated.

### Cell migration and invasion assay

Cell migration and invasion potential was assessed by wound healing and transwell assays, respectively. For transwell assay, cells were trypsinized and 1 × 10^5^ cells in 100 *μ*l of serum-free RPMI-1640 medium were plated into the upper chamber. RPMI-1640 medium (500 *μ*l) supplemented with 20% FBS was added to the lower chamber. After culturing for 22 h, cells that had invaded the lower chamber were fixed with methanol and stained with 0.1% crystal violet. The number of invaded cells was observed by using an inverted microscope (magnification × 200) and calculated by counting five random views. For wound healing assay, cells were trypsinized and seeded in 6-well plates, and 12 h later an artificial wound was created by using a 200 *μ*l pipette tip. The wound was observed after 24 h and imaged under a microscope. The fraction of cell coverage across the line was measured for the migration rate.

### Tumorigenesis and metastasis assays

The 5-week-old Female BABL/c athymic nude mice were obtained from the Beijing Vital River Laboratory Animal Technology Co., Ltd (Beijing, China). For tumorigenesis analysis, 1 × 10^6^ cells stable knockdown of lncRNA XIST (sh-XIST) or negative control cells (sh-NC) were inoculated into the right flanks of each mouse. Tumor diameter (mm) was measured every week, and the tumor volumes were calculated using the formula *V*=(shortest diameter)^2^ × (longest diameter) × 0.5. After 35 days, the mice were killed and the tumors were exercised. For metastasis analysis, 2 × 10^6^ cells were inoculated into the tail vein of each mouse, and after 6 weeks the mice were killed and the lung and liver were exercised and paraffin embedded. Consecutive sections (4 *μ*m) were made using the tissues, and stained with hematoxylin–eosin. The micrometastases in the lungs and livers were evaluated by a dissecting microscope. All the animal experiments were performed according to the National Institutes of Health animal use guidelines on the use of experimental animals.

### Vector construction and luciferase reporter assay

The 3′-end fragment from lncRNA XIST containing the predicted miR-200b-3p-binding site was amplified using PCR and subcloned into a pmirGLOluciferase Target Expression Vector (Promega, Madison, WI, USA) to form the XIST wild-type (pmirGLO-XIST-wt) vector. The mutated miR-200b-3p-binding sequence was constructed that was named as pmirGLO-XIST-mt vector. The HEK 293T cells were co-transfected with PrirGLO, pmirGLO-XIST-wt, prirGLO-XIST-mt and miR-200b-3p mimics or negative control using Lipofectamine 2000, and the relative luciferase activity was measured using the Dual-Luciferase Reporter Assay Kit (Promega) after 48 h.

### Western blot analyses

Western blot analysis was performed using a method described previously.^36^ The primary antibodies used in this study were as follows: ZEB1 (ABE596), GAPDH (2118 L), E-cadherin (3199 S) and N-cadherin (14215 S).

### Statistical analyses

All statistical analyses were performed using the SPSS (version 16.0, SPSS Inc., Chicago, IL, USA) or GraphPad Prism 5.0 (GraphPad Software, Inc, CA, USA). The results were expressed as mean±S.D. Survival analysis was evaluated using the Kaplan–Meier method and assessed using the log-rank test. Student’s *t*-test or one-way ANOVA was used to analyze the *in vitro* and *in vivo* data. A *P*-value of <0.05 was considered to be statistically significant.

## Publisher’s Note

Springer Nature remains neutral with regard to jurisdictional claims in published maps and institutional affiliations.
