# Supplementary material for: Long noncoding RNA XIST expedites metastasis and modulates epithelial–mesenchymal transition in colorectal cancer
Source: Cell Death Dis. 2017 Aug 24;8(8):e3011–. doi: 10.1038/cddis.2017.421 (PMC5596599; doi:10.1038/cddis.2017.421)
Supplement: Supplementary Table S1 [file cddis2017421x1.docx]

**Supplementary Table S1** The correlation between clinicopathological parameters and lncRNA XIST expression levels in 115 CRC patients

| Variables | n | Low XIST expression (%) | High XIST expression (%) | *P* value |
| --- | --- | --- | --- | --- |
| Age |  |  |  | 0.449 |
| <60 | 81 | 39(67.2) | 42(73.6) |  |
| ≥60 | 34 | 19(32.8) | 15(26.4) |  |
| Gender |  |  |  | 0.379 |
| Male | 70 | 33(56.2) | 37(64.9) |  |
| Female | 45 | 25(43.8) | 20(35.1) |  |
| Tumor size |  |  |  | 0.001^a^ |
| <5cm | 43 | 30(56.8) | 13(22.8) |  |
| ≥5cm | 72 | 28(3.2) | 44(77.2) |  |
| Histological grade |  |  |  | 0.018^a^ |
| Well | 23 | 13(22.4) | 10(17.5) |  |
| Moderate | 34 | 23(39.6) | 11(19.2) |  |
| Poor and others | 58 | 22(39.0) | 36(63.3) |  |
| Tumor location |  |  |  | 0.155 |
| Right colon | 65 | 29(50.0) | 36(63.1) |  |
| Left colon/rectal | 50 | 29(50.0) | 21(36.9) |  |
| Lymph node invasion |  |  |  | 0.076 |
| Absent | 64 | 37(63.7) | 27(47.3) |  |
| Present | 51 | 21(36.3) | 30(52.7) |  |
| Distant metastasis |  |  |  | 0.001^a^ |
| Absent | 42 | 30(51.7) | 12(21.0) |  |
| Present | 73 | 28(48.3) | 45(79.0) |  |
| TNM stage |  |  |  | 0.006^a^ |
| I-II | 47 | 31(53.4) | 16(28.0) |  |
| III-IV | 68 | 27(46.6) | 41(72.0) |  |

^a^*P* **<** 0.05, Chi-square test.
