# Supplementary material for: Long noncoding RNA XIST expedites metastasis and modulates epithelial–mesenchymal transition in colorectal cancer
Source: Cell Death Dis. 2017 Aug 24;8(8):e3011–. doi: 10.1038/cddis.2017.421 (PMC5596599; doi:10.1038/cddis.2017.421)
Supplement: Supplementary Table S2 [file cddis2017421x2.docx]

**Supplementary Table S2** Univariate and multivariate analyses of various potential prognostic factors in 115 CRC patients

| Factors | Univariate analysis | |  | Multivariate analysis | |
| --- | --- | --- | --- | --- | --- |
|  | HR^b^(95%CI^c^) | *P* |  | HR^b^(95%CI^c^) | *P* |
| Age | 1.03 (0.87-1.15) | 0.342 |  | - | - |
| Gender | 1.12 (1.03-1.45) | 0.215 |  | - | - |
| Tumor size | 1.23 (1.01-1.46) | 0.089 |  | - | - |
| Differentiation | 1.02 (0.78-1.42) | 0.112 |  | - | - |
| Lymph node invasion | 1.43 (1.12-1.97) | 0.047 ^a^ |  | 0.87 (0.56-1.36) | 0.123 |
| Distant metastasis | 1.56 (1.13-2.08) | 0.025^a^ |  | 1.35 (1.15-2.56) | 0.033^a^ |
| TNM stage | 1.47 (1.23-2.22) | 0.021^a^ |  | 1.15 (1.08-2.36) | 0.116 |
| lncRNA XIST expression | 2.76 (1.12-2.79) | 0.010^a^ |  | 1.29 (1.11-1.96) | 0.039^a^ |

^a^*P* **<** 0.05.

^b^HR, hazard ratio.

^c^CI, confidence interval.
