# Supplementary material for: Long noncoding RNA XIST expedites metastasis and modulates epithelial–mesenchymal transition in colorectal cancer
Source: Cell Death Dis. 2017 Aug 24;8(8):e3011–. doi: 10.1038/cddis.2017.421 (PMC5596599; doi:10.1038/cddis.2017.421)
Supplement: Supplementary Table S3 [file cddis2017421x3.docx]

**Supplementary Table S3** Primers used in the paper were listed

| **Gene** | **Primer** | **Sequence(5′-3′)** |
| --- | --- | --- |
| **Primers for qRT-PCR** | |  |
| lncRNA XIST | forward | 5’-CTCTCCATTGGGTTCAC-3’ |
|  | reverse | 5’-GCGGCAGGTCTTAAGAGATGAG-3’ |
| N-cadherin | forward | 5’-GGTGGAGGAGAAGAAGACCAG-3’ |
|  | reverse | 5’-GGCATCAGGCTCCACAGTG-3’ |
| Vimentin | forward | 5’-GAGAACTTTGCCGTTGAAGC-3’ |
|  | reverse | 5’-GCTTCCTGTAGGTGGCAATC-3’ |
| Snail | forward | 5’-CCTCCCTGTCAGATGAGGAC-3’ |
|  | reverse | 5’-CCAGGCTGAGGTATTCCTTG-3’ |
| Slug | forward | 5’-GGGGAGAAGCCTTTTTCTTG-3’ |
|  | reverse | 5’-TCCTCATGTTTGTGCAGGAG-3’ |
| E-cadherin | forward | 5’-TGCCCAGAAAATGAAAAAGG-3’ |
|  | reverse | 5’-GTGTATGTGGCAATGCGTTC-3’ |
| α-catenin | forward | 5’-AGCGAATTGTGGCAGAGTGT-3’ |
|  | reverse | 5’-GTCTACGCAAGTCCCTGGTC-3’ |
| β-catenin | forward | 5’-ACAACTGTTTTGAAAATCCA-3’ |
|  | reverse | 5’-CGAGTCATTGCATACTGTCC-3’ |
| CD44 | forward | 5’-TTGCAGTCAACAGTCGAAGAAG-3’ |
|  | reverse | 5’-CCTTGTTCACCAAATGCACCA-3’ |
| Oct4 | forward | 5’-CTTGCTGCAGAAGTGGGTGGAGGAA-3’ |
|  | reverse | 5’-CTGCAGTGTGGGTTTCGGGCA-3’ |
| CD133 | forward | 5’-TGGATGCAGAACTTGACAACGT-3’ |
|  | reverse | 5’-ATACCTGCTACGACAGTCGTGGT-3’ |
| CD24 | forward | 5’-TGAAGAACATGTGAGAGGTTTGAC-3’ |
|  | reverse | 5’-GAAAACTGAATCTCCATTCCACAA-3’ |
| CD166 | forward | 5’-TCCTGCCGTCTGCTCTTCT-3’ |
|  | reverse | 5’-TTCTGAGGTACGTCAAGTCGG-3’ |
| CD155 | forward | 5’-TATCTGGCTCCGAGTGCTTGCC-3’ |
|  | reverse | 5’-ATCATAGCCAGAGATGGATACC-3’ |
| SOX2 | forward | 5’-GCCGATGTGAAACTTTTGTCG-3’ |
|  | reverse | 5’-GGCAGCGTGACTTATCCTTCT-3 |
| Nanog | forward | 5’-AATACCTCAGCCTCCAGCAGATG-3’ |
|  | reverse | 5’-TGCGTCACACCATTGCTATTCTTC-3’ |
| ZEB1 | forward | 5’-TAAAGTGGCGGTAGATGGTA-3’ |
|  | reverse | 5’-ACTGTTTGTAGCGACTGGATT-3’ |
| GAPDH | forward | 5’-TGCACCACCAACTGCTTAGC-3’ |
|  | reverse | 5’-GGCATGGACTGTGGTCATGAG-3’ |
| U6 | forward | 5’-CTCGCTTCGGCA GCACA-3’ |
|  | reverse | 5’-AACGCTTCACGAATT TGCGT-3’ |
